# A Patient With (Initially) Non-Persistent Vertigo – A Posterior Circulation Stroke Case

**DOI:** 10.7759/cureus.21468

**Published:** 2022-01-21

**Authors:** Ana Costa, Olinda Miranda, Ana Cerqueira, Carlos Fernandes, Jorge Cotter

**Affiliations:** 1 Internal Medicine, Hospital da Senhora da Oliveira, Guimarães, PRT

**Keywords:** vertebrobasilar insufficiency, posterior circulation syndrome, posterior circulation stroke, endovascular angioplasty, vertebrobasilar ischemia

## Abstract

Posterior circulation strokes are responsible for about 20-25% of all ischemic strokes. Recognition and diagnosis of posterior circulation strokes or transient ischemic attacks is more difficult than that of other stroke types, being frequently misdiagnosed in acute setting/emergency evaluation - up to more than three times as often as anterior circulation strokes.

In accordance with the guidelines in effect at the time of this case presentation, thrombolysis and medical therapy are the mainstay treatment. Mechanical thrombectomy is emerging as a treatment option in posterior vessel occlusion but randomized clinical trials are still lacking.

## Introduction

The posterior circulation comprises both vertebral arteries (VAs), the basilar artery, and the intracranial vessels that they give rise to. Together, these arteries supply the brainstem, cerebellum, medial and postero-lateral thalamus, occipital lobes, and sometimes parts of the medial temporal and parietal lobes [1-3].

Posterior circulation strokes may pose a challenge in diagnosis presenting with symptomatology that can be somewhat more subtle than anterior circulation strokes. Typically, the most common symptoms include dizziness and syncope (47%), unilateral limb weakness (41%), dysarthria (31%), headache (28%), and nausea or vomiting (27%). The most frequent signs are unilateral limb weakness (38%), gait ataxia (31%), unilateral limb ataxia (30%), dysarthria (28%), and nystagmus (24%) [1-3].

In this case, the diagnosis was made after the presence of ischemic cerebellar injury in brain computed tomography (CT), although symptoms were present a few weeks early. The patient’s clinical and imaging evaluation also posed a challenge.

## Case presentation

A 50-year-old man presented to the emergency department (ED) with disequilibrium, dizziness, and visual changes. According to the patient, his complaints started three weeks prior to the admission - episodes of blurred vision, imbalance, and tinnitus, which lasted for few seconds to minutes, self-limited with spontaneous resolution. He was evaluated at the ED, had a head computed tomography (CT) - which was normal - and has been discharged with therapy for vertigo. In the following weeks, his complaints became more persistent and disabling, adding complaints of occasional diplopia, which motivated another evaluation at the ED and also with his family physician, who requested a brain magnetic resonance imaging (MRI) and a neck vessels Doppler echography (done five days previous to admission), both reported normal (results observed in ED).

In his last visit to the ED, the patient complained about dizziness, with orthostatic intolerance, imbalance, ataxia, and tinnitus. He also mentioned inability to write.

His past medical conditions included hypertension, dyslipidemia, smoking (28 pack-year), and alcohol consumption (estimated 90 g/day). He had a minor surgery procedure - excision of cyst on his back, the day before the onset of his complaints.

**Figure 1:**
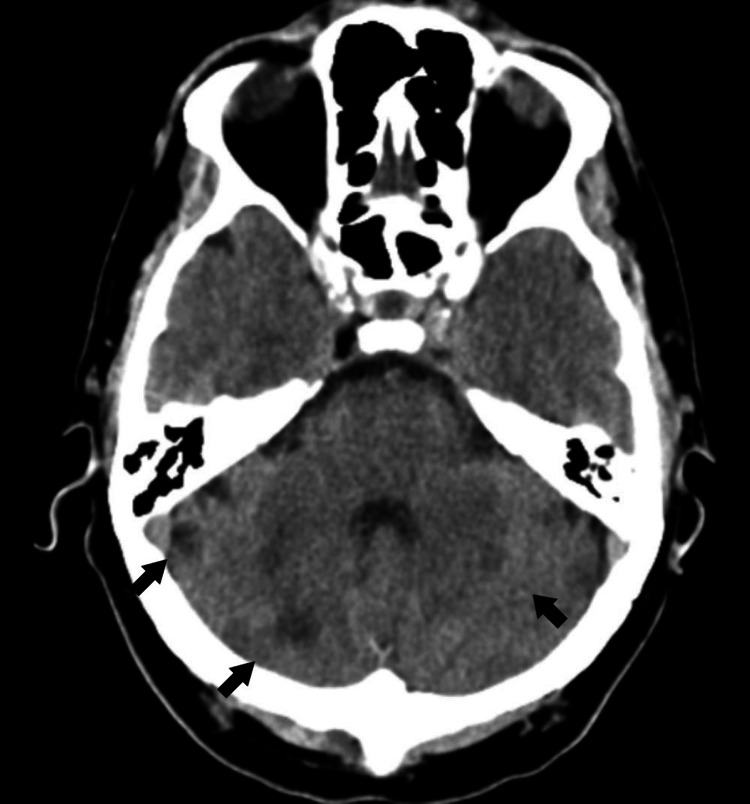
Admission brain-CT showing multiple and bilateral cerebellar ischemic lesions (arrows). CT, computed tomography.

At admission, his physical examination was described as normal although gait was not tested. A brain CT showed multiple and bilateral cerebellar infarcts (Figure 1).

Due to this finding, a transthoracic echocardiogram was done, which showed no evidence of embolic source.

The patient was admitted with the diagnosis of cerebellar infarct.

On day 1, the patient presented with a left rapid-phase nystagmus, which had changed during the time of his hospital stay to a right rapid phase and lately to a bilateral nystagmus. He also presented with dysmetria initially only on the left side, which also changed in the same way as the nystagmus (initially unilateral, progressing to bilateral). He maintained complaints of dysgraphia. On day 3, he presented with dysarthria.

The patient had a transesophagic echocardiogram done (day 2), which was reported as normal (excluding cardioembolic events and patent foramen ovale). His blood work is presented in Table 1.

**Table 1:** Laboratory results. HDL, high-density lipoprotein; LDL, low-density lipoprotein; HbA1c, hemoglobin A1c.

Test result		Reference value
Total cholesterol	157	<200 mg/dL
HDL cholesterol	51	40-60 mg/dL
LDL cholesterol	87	<130 mg/dL
Triglycerides	95	30-150 mg/dL
HbA1c	5.8	3.8-5.9%
Syphilis screening	<0.1	Negative <0.9 (ratio)
Folic acid	7.9	3.1-17.5 ng/mL
Cyanocobalamin	665	211-911 pg/mL

**Figure 2:**
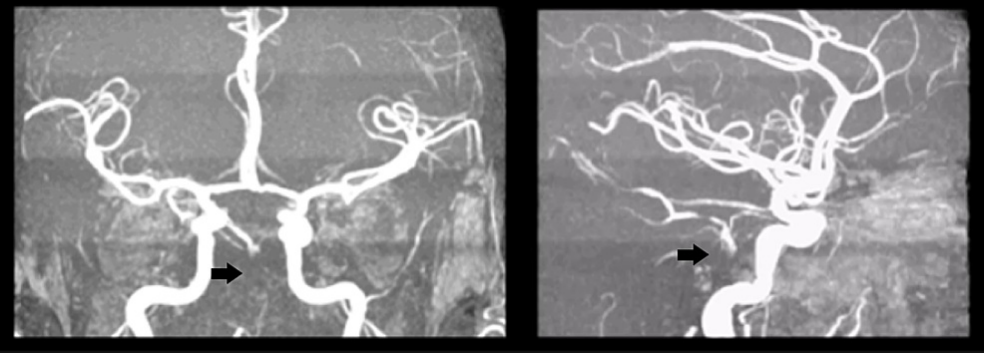
Angio-MRI with no flux in basilar artery. MRI, magnetic resonance imaging.

An angio-MRI (day 4) revealed total basilar artery occlusion, with no worsening of the infarct zones (Figure 2).

At this time, clopidogrel was added to acetylsalicylate acid, starting double anti-aggregation therapy. A neuroradiology consultation was obtained and it was decided to keep the patient under surveillance.

**Figure 3:**
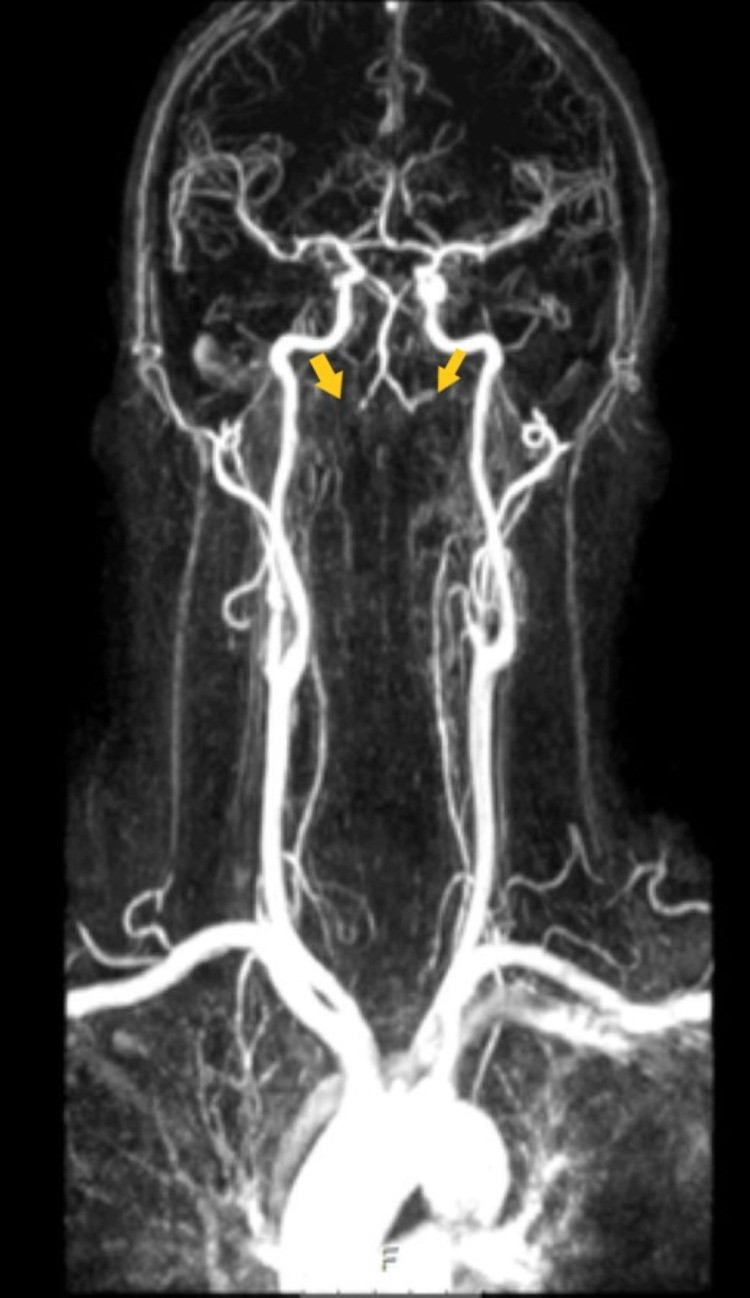
Brain angio-CT with bilateral vertebral occlusion. CT, computed tomography.

Four days later, the patient had a brain angio-CT including supra-aortic vessels done, which showed a patent basilar artery, but bilateral VA occlusion (Figure 3), raising questions about its etiology: thrombus (normal transthoracic and transesophagic echocardiogram), arterial dissection or vasculitis (normal blood tests, including sedimentation rate and autoimmunity), atherosclerosis (unstable plaque). It was decided to maintain vigilance and to start physical rehabilitation.

**Figure 4:**
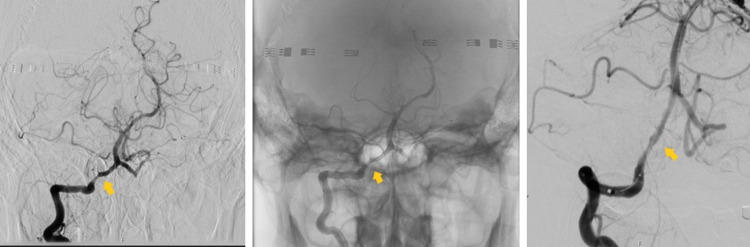
Angiography and endovascular treatment. The arrows point to right vertebral artery stenosis. In the middle and right images we see a guiding catheter passage.

A few days later, and still under double anti-aggregation therapy, the patient developed peripheral facial palsy. A brain angio-CT showed an occluded left VA, with a patent right VA but with an occluded ipsilateral posterior inferior cerebellar artery (PICA). After consultation with a neuroradiology intervention team, the patient was transferred to the unit and underwent angiography that revealed atheromatous segmental stenosis of right V4 segment; atheromatous stenosis of right VA ostium of 50%; and distal left VA segment with retrograde flow, with total occlusion of the artery. Probable atherothrombotic etiology was assumed. A stent was placed in the right V4 segment with residual stenosis of 30%. There was no evidence for atherosclerotic/atherothrombotic disease in both carotid arteries (Figure 4).

After the procedure, the patient recovered from his nystagmus, regained balance, and was able to write/draw again although with limitation. At the time of discharge, he still had the facial palsy but it was resolving. He was walking on his own, with enlarged base. He was kept on double anti-aggregation and statin.

He had follow-up medical consultation, presenting in one-year follow-up with no physical limitations, besides a minor dysgraphia, but with sluggish thinking.

## Discussion

Vertebro-basilar insufficiency refers to a condition where posterior circulation is affected and there is hypoperfusion in the posterior fossa. It comprehends vast clinical presentation syndromes, which can make the diagnosis difficult. The main symptoms overlap with many benign conditions such as paroxysmal benign postural vertigo, labyrinthitis, and neuritis. It is more frequent in men after the fourth decade, and hypertension, diabetes, dyslipidemia, obesity, and smoking are high-risk factors [2,3].

Basilar artery occlusion, while rare (1% of all strokes), can cause brainstem or thalamic infarction, which can result in severe syndromes such as complete limb and facial paresis with preserved consciousness (the “locked-in” syndrome), reduced consciousness, and oculomotor abnormalities (from midbrain and bilateral thalamic damage, as part of the “top of the basilar” syndrome), coma, and cardiorespiratory disturbances, depending on the site of occlusion [2-5]. It has an extremely poor prognosis but can benefit from intra-arterial thrombolysis or mechanical thrombectomy (clot retrieval) even 24 hours after symptom onset [6]. 

Like anterior circulation ischemic stroke, the main cause is atherosclerosis, followed by embolism, vertebral dissection, and aneurysm [1,2]. In this case, atherothrombotic disease was found in the right VA but not in any other evaluated vascular territory. 

Endovascular treatment is a promising approach with improved outcomes in several studies and has been graded IIb recommendation in the stroke guideline 2019. However, more data are needed [5].

In this case report, our patient had a first episode with ischemic lesions confirmed in his brain-CT with a documented “no-flow” in the basilar artery. However, his symptoms fluctuated and ultimately he presented with a facial palsy with a documented PICA occlusion de novo. The intervention timing exceeded the guideline recommendation, but the patient had a great recovery of his neurological deficits.

## Conclusions

Posterior circulation strokes still pose a diagnostic challenge and are often misdiagnosed. There is still debate regarding the best treatment options (best medical care vs endovascular treatment vs both), with different results in recent studies. In this case, the patient underwent endovascular treatment after the recommended period and in the presence of established ischemic lesions, mainly due to clinical deterioration. Due to their singularity, posterior circulation strokes' treatment should be appropriate for each situation.
